# Progress in Medicine

**Published:** 1895-06-29

**Authors:** 


### Progress in Medicine.

### INFECTIVE DISEASES.

**Typhoid.**—Gangrene occurs not infrequently as a complication, according to L. H. Mettler. Thus Keen records forty-three cases and Barchoud twenty. It is found in males more often than in females, usually during epidemics amid depressing circumstances.[1] Neither weakened circulation nor endocarditis appears to be the cause, but rather "an obliterative arteritis lighted up by a virulent toxœmia" occurring during the third week or at the beginning of convalescence. The prognosis, owing to the state of the patients, is frequently unfavourable. Drewett[2] relates an instance in a child where indeed after amputation of the leg recovery followed. He believed the cause to be an embolus. Chauffard had twelve typhoid cases out of twenty-five presenting various abscesses apparently due to the numerous baths given in impure water. The staphylococcus aureus found in many of the abscesses could be obtained from the water previous to its use. Achard had met with these abscesses only when the same tub was used for several patients. They were till lately thought to be due to infection from the intestines, but appear rather to be produced by external causes when frequent baths are given. These abscesses must be distinguished from suppuration really due to the bacillus. Thus Swiezynsky met with an abscess under the deltoid around the shoulder joint, in which the typhoid bacillus alone was present; nor are such cases rare.[3] A diabetic patient of Manchot's afforded an instance of septicæmia from the staphylococcus flavus, which showed symptoms closely simulating typhoid.[4] There was an enlarged spleen, a roseolar eruption, and a typical temperature, but typhoid lesions were absent at the autopsy. Banti,[5] too, relates a group of cases where only the rash was absent from the usual group of symptoms, but the disease proved not to be typhoid. On the other hand, he mentions a case of true typhoid septicæmia with no abdominal symptoms or rash.

Ulnar paralysis following typhoid in a boy is noted by Wolf. It came on without pain unexpectedly after convalescence.[7] J. A. Amyot draws attention to the changes during typhoid in the liver. He found a number of small nodules present in all the cases he examined, made up of unstained granular protoplasm.[8] There were also areas of capillary dilatation where only the nuclei were left in the hepatic cells. In some cases atrophic and fatty changes were also met with.

The blue spots noticed by Trousseau and other writers have been seen by A. V. M. Anderson in eight out of 915 cases, varying in size from a pea to that of a threepenny piece. He could find no support for the view that they result from pediculi, and as they occur over subcutaneous veins there is reason to think they depend on some exudations from the vessels.[9] Nor have they any clinical significance, being found in both mild and severe attacks at various stages. Murchison gave 100 days as the longest duration of fever in typhoid, but A. R. Parsons recently brought foward an instance of fever extending to 290 days and ending in complete recovery. There were hardly any complications. N. Falkiner related another where pyrexia lasted 119 days, while hæmorrhage and diarrhœa persisted during the greater part of the time.[10] Dr. Parsons also showed laryngeal ulcers which appeared to be typical typhoid ones due to the bacillus.[11] In a recent number we noticed the actual discovery of bacilli in similar ulcers by P. W. Williams. Membranous sore throat in typhoid is discussed by W. G. Smith.[12] In his case the membrane was due to streptococci only, though the symptoms were those of true diphtheria. The complication is certainly uncommon, and the need of a bacterial examination is evident.

In connection with the appearance of gangrene mentioned above, the effect of the specific virus in causing aortitis should not be overlooked. Potain regards it as the cause of serious arterial failure in later life, though the immediate prognosis is very good, and there may be hardly any symptoms.[13] Enlargement of the area of cardiac dulness and quickened pulse may be all that is noticed. If there is any fear of this complication we should avoid over-feeding or stimulating the circulation in any way. Iodine internally and blistering may be of use in treatment.

Galliard[14] describes the desquamative erythema occasionally occurring during convalescence, or in earlier stages. It usually commences as symmetrical patches of papules, which change to a scarlatina-form rash over the greater part of the skin, which finally peels off in large flakes. The temperature rises again, and the condition is one of considerable danger to the patient. Tonics and alkalies should be substituted for intestinal antiseptics, and soothing ointments may relieve the irritation. It appears to be a true secondary infection, but the exact ætiology is uncertain.

The prodromal stage of typhoid is the subject of a very interesting paper by Hanot.[15] The malaise may, he thinks, last even two or three weeks before the febrile period commences, and may be mistaken for dyspepsia, malaria, tuberculosis, syphilis, influenza, and other complaints. Indeed, an entirely apyretic form of typhoid has been occasionally observed, of which he describes two fatal cases. Besides headache, diarrhœa or prostration, there may be sore throat, pneumonia, or mania in this prodromal stage. The difficulty of diagnosis should teach caution in excluding an early stage of typhoid.

In another paper on the later stages he notices as occasional complications acute fatty change and miliary abscesses in the kidney,[16] the delirium of convalescence, and broncho-pneumonia. We may often find polyuria, slow pulse, and morning subnormal temperatures of value in marking the beginning of true convalescence, as distinguished from an apyretic period preceding a relapse.

To shorten the duration of typhoid E. H. Embley recommends[17] the use of B. Naphthol dissolved by heat in olive oil, and given as an emulsion. It thus produces far better results than when in the solid form; and A. M. Anderson claims boldly that under his salol-bismuth treatment every uncomplicated case, if treated early, becomes apyretic by the fifteenth day. In his paper[18] he notices a curious source of infection from the use of a contaminated enema syringe. This might frequently happen with careless nursing, and, indeed, the clinical thermometer does occasionally convey disease when imperfectly disinfected. Dreschfeld[19] remarks that sometimes the sputum and urine contain bacilli and need disinfection, and also that vegetables, when contaminated by water, and even flies may be bearers of the typhoid germs. The report on sewer air which was presented to the London County Council shows that it rarely, if ever, contains organisms derived from the sewage, such as the bacilli coli, and cannot, therefore, directly communicate typhoid. "The less the ventilation the fewer germs are found in the sewer air," but still it may lower vitality and the resistance to infection. Though sewage itself may carry the germs to the subsoil through leaks, it is not a medium in which the bacillus can long survive.[20] The bacillus coli is by Kellogg still regarded as possibly a variety of Eberth's bacillus,[21] and as capable of producing typhoid by itself. It is, besides, specially hardy and resistant. Gilbert, in a long account of its properties, denies this view, but considers that there are some half-dozen different varieties of it; and though during health it does no injury to its host, yet in disease of the liver or kidneys and other lesions it is the cause of many of the most common symptoms. Uræmia is often a coli-bacillary intoxication, so too is frequently the "typhoid state."

When its virulence is great it often sets up diarrhœa or intestinal paralysis, and travelling through the system it produces pneumonia, cystitis, and a very long list of other affections.[22] That the bacillus is really the agent in these cases is shown by the possibility of reproducing a given lesion by injecting into animals the bacillus from, say, endocarditis. Pfeiffer shows that there is an important diagnostic mark between the bacillus coli and typhosus in the fact that the serum of animals immunised against typhoid is fatal to cultures of B. typhosus, but not to those of B. coli.[23] The typhoid poison is contained chiefly in the bodies of the bacilli and not in the filtered cultures. Chantemesse, too, and many others deny the production of typhoid by the bacillus coli as contrary to all known facts.[24] The report of G. Sims Woodhead and G. E. C. Wood on the efficiency of table filters went to show the utter uselessness of most common filters as a protection against water-borne disease.[25] Typhoid and other baccilli passed through them in large numbers. An exception must be made in favour of the Pasteur-Chamberland, the Berkefeld, and the Porcelaine d'Amiant makes. It is strange to notice how devotedly the public believe the statements of many makers that certain filters completely remove the germs of disease, just as they do the assertion that prolonged infusion extracts an excess of tannin from tea, in spite of clear evidence to the contrary. In fact, bad filters actually increase the danger of impurity in water, though they improve its taste and appearance. It is fair to say that two firms have since brought forward some little evidence in favour of their special productions, but the number of really efficient filters is very small, if, indeed, any can long be trusted.[26] A most prolix correspondence took place at the end of the year on the conveyance of typhoid through oysters, and a great number of cases where they seemed to be the only possible channel for infection were narrated by Sir W. Broadbent[27] and others,[28] just at a time when a brilliant investigation had traced to the same source a severe outbreak in Wesley University, Connecticut.[29] Here the only food common to all the sufferers turned out to be some oysters eaten at certain club feasts. They had been laid down to fatten in a creek just where the sewage from two cases of typhoid ran down. Cases in other places of persons who had eaten the same oysters then came to light. The evidence as to the time typhoid bacilli survive in sea water is, according to Frankland, uncertain,[30] but the drachm or so of water which is held in the mantle of an oyster may have been infected immediately before it was taken from the sea. Giaxa, from the Naples Marine Laboratory, however, found the bacilli alive nine days after being placed in sea water, and the report to the County Council did not prove their rapid death, but only that sewage is unfavourable to their growth.[31] A series of reports on the chief oyster beds shows sewage contamination in some places, but on the whole is reassuring.[32] Still, the writer concludes, we cannot doubt that it is absolutely necessary to get rid of sewage from all estuaries where oysters are cultivated. Among outbreaks of typhoid, the Paisley[33] one, of 859 cases, showed that the water supply was at least doubtful. A small one in New Jersey[34] was referred to milk, and another to an infected bakery and well[35]; one of 150 cases at Windsor, U.S., arose from a contaminated reservoir,[35] while Washington appears to derive its constant infection from numerous wells in a soil saturated with fœcal matters.[36] Havre, too, seems to be the great focus in Northern France for typhoid; and while Gibert refers the constant infection to the infected subsoil, Brouardel combats his views, and asserts that the real source is a contaminated water supply. The time between the contamination and a recent outbreak was, however, eight or nine months, which is a long period for the survival of the bacillus in earth or water.[37] Welply pleads again for the inspection of co-operative milk factories. The discovery of pathogenic germs in cheese and butter, and the known conveyance of typhoid in cream, renders this subject an important one.[38]

As to treatment, Henry, of Melbourne, advocates infusions of rice, barley, and oatmeal, with malt extract and fruit juice, in preference to milk or broth. Fisk[40] gives milk alone, with frequent four-minim doses of turpentine in castor oil and bismuth! Kœnig gives one or two drop doses of guiacol every two hours, and $\frac{1}{24}$ to $\frac{1}{10}$ grains of calomel three or four times daily till some diarrhœa follows. Cold sponging when necessary, small repeated doses of alcohol, and a liquid diet are also advocated by him.[41] The need of frequent cleansing of the naso-pharynx by Dobell's or some other solution during fevers, to prevent local inflammation, is the subject of papers by W. C. Phillips and C. C. Rice.[42] Intestinal paresis is often a serious and troublesome complication. For its treatment Fernet[43] recommends the continuous current of about six milliampères daily, one pole being placed over the loins, and the other moved over the large intestine. Lactophenin as an antipyretic was used by Strauss[44] without bad effects in twenty-five cases where bathing was impracticable; and Brill reports unfavourably of guiacol used externally, since, when enough is used to bring down the temperature, serious collapse follows.[45] In smaller amounts—ten to twenty drops—it is, however, useful as an anti-neuralgic. Phenacetin is favoured by Shattuck[46] as an alternative to baths. He allows a rather wide range of food if the patient can digest it, even eggs, custards, and scraped meat, besides milk, with a plentiful supply of water to eliminate soluble poisons.

Seymour Taylor speaks of the good results seen in St. Thomas's Hospital from giving grey powder three or four times daily. He does not stop diarrhœa unless more than four motions a day are passed, and for constipation advises warm water enemata with a little olive oil.[47]

Few reports of serum inoculation have appeared. Girard,[48] however, narrates a case where the temperature had kept near 104 degrees for two weeks in spite of various applications, and death seemed imminent. He then obtained eight c.c. of serum by a blister from another patient who had been free from fever for a week. This was injected under the skin of the abdomen. The temperature gradually fell and convalescence ensued. Kraus, of Vienna,[49] used sterilised cultures of blue pus to render guinea pigs immune, and employed their serum on twelve typhoid patients without much success. It will be recollected that Sanarelli had somewhat similar results. A curious effect has been noticed during the removal of convalescents by ambulance from one hospital to another near London. Albuminuria occurred in about 3 per cent. in large amounts, but generally disappeared as rapidly,[50] and the same thing was noticed when removal was necessary during the acute stage of the fever. The cold bath controversy has brought out statistics from the Birmingham General Hospital where 304 patients were treated on the expectant plan with a mortality of 51 or 16·8 per cent.[51] Osler's cases treated by cold baths numbered 196, and the mortality was 7·1 per cent. The Birmingham patients came into the hospital at a later period of the disease than Osler's did on an average, and, of course, there may have been a difference in the virulence of the epidemics. Still the results confirm numerous previous statistics. An analysis of the results of 1,000 baths in the Massachusetts General Hospital gives an average drop of $\frac{2}{5}$ of a degree for sponge baths, and $2\frac{2}{5}$ for each full bath registered half an hour after either.[52] The fall was maintained much longer, too, after the full baths, but the latter were very disagreeable to the patients. Sponge baths relieved cerebral symptoms as well as the others, but no conclusions as to the the comparative mortality could be drawn. It is much to be wished that statistics of a large number of cases where the temperature had been kept down by the safer antipyretics to Brand's maximum of 102·5 degrees were published for comparison with those of the cold bath method. Bedford Brown[53] employs a thin poultice of a temperature rather under 70 degrees over the whole chest and abdomen. This he applies every hour when there is hyperpyrexia. When cerebral symptoms appear, it is applied to the head and spinal cord, and the results are said to be gradual and most effectual without the difficulties and discomforts of the bath. The continuous bath system of Dr. Barr was employed by F. J. Smith[54] in a case where the temperature rose to 105 whenever the patient was removed from the water. She had been comatose at first, and had a slight hæmorrhage. She remained in the water for 32 days in spite of slight pneumonia and a sacral abscess. Both complications rapidly passed away, and she made a perfect recovery. The water was kept at about a temperature of 95 degrees, and the patient seemed to thoroughly enjoy her long bath. Vogl,[55] of Munich, gives his mortality at 5·1 per cent, which was raised to 7·3 during the influenza. He found that antipyrin produces only a short fall of temperature, diarrhœa is not checked, and the renal secretion is lessened, while with baths the reverse is the case.

We may conclude this summary by a few words on the success of the French in eliminating typhoid from their army.[56] The cases have fallen from over 14 to less than 6 per thousand in eight years through the use of the Pasteur filters, while in our own army it is still 9·14 per thousand. The Cherbourg garrison, which had an annual average of 114·5 cases, have now only 3. In India the mortality from typhoid among the young troops is frightful, and shows little sign of improvement.

1 N. Y. Med. J., Mar. 9, 1895. 2 B. M. J., Feb. 23. 3 Am. J. Med. Sc, Mar. 4 Med. Week, Nov. 23. 5 Am. J. Med. Sc., Jan. 7 Am. J. Med. Sc., Mar. 8 Lanc., Feb. 16. 9 N. Y. Med. J, Oct. 20. 10 Lanc., Apr. 6. 11 Lanc., Jan. 17. 12 Practit., Feb. 13 Med. Week, Nov. 9. 14 Med. Week, Oct. 26. 15 Med. Week, Nov. 23. 16 Med. Week, Mar. 1. 17 Birmingh. Med. Rev., Nov. 18 Glasgow Med. J., Nov. 19 Lanc., Ap. 20. 20 Lanc., Feb. 9. 21 Med. Rec., Oct. 27. 22 Med. Week, Jan. 11. 23 Am. J. Med. Sc., Feb. 24 Med. Week, Sept. 21. 25 B. M. J., Nov. 10, et seq. 26 B. M. J., Mar. 2. 27 B. M. J., Jan. 12. 28 B. M. J., Jan 19. 29 Med. Rec., Dec. 15. 30 Nature, Feb. 28. 31 B. M. J., Feb. 16. 32 B. M. J., Jan. 19, et seq. 33 B. M. J., Oct. 27. 34 Med. Rec., Feb. 9. 35 Med. Rec., Nov. 24. 36 Med. Rec., Dec. 22. 37 Dublin J. Med. Sc., Oct. 38 Lanc., Nov. 10. 39 B. M. J., Ap. 6. 40 Ther. Gazette, Dec 15. 41 Ther. Gazette, Nov. 15. 42 Med. Rec., Feb. 9. 43 Med. Week, Jan. 4 44 B. M. J., Dec. 8. 45 B. M. J., Dec. 22. 46 Am. J. Med. Sc., Ap. 47 Clin. J., Jan. 23. 48 J. Am. Med. Assoc., Oct. 27. 49 Med. Rec., Mar. 23. 50 Lanc., Mar. 16. 51 Birmingh. Med. Rev., Dec. 52 Ther. Gazette, Jan. 15. 53 Ther. Gazette, Dec. 15. 54 Clin. J., Jan. 23. 55 B. M. J., Ap. 27. 56 B. M. J., Ap. 27.

